# Quality cancer survivorship care: a modified Delphi study to define nurse capabilities

**DOI:** 10.1007/s11764-025-01804-6

**Published:** 2025-05-03

**Authors:** Gemma McErlean, Heidi Hui, Fiona Crawford-Williams, Nicolas H. Hart, Meinir Krishnasamy, Bogda Koczwara, Thomas Walwyn, Mahesh Iddawela, Rebecca McIntosh, Raymond J. Chan, Michael Jefford

**Affiliations:** 1 School of Nursing, University of Wollongong, Sydney, NSW Australia; 2 Centre for Research in Nursing and Health, St George Hospital, Sydney, NSW Australia; 3 Health Innovations Research Centre, Faculty of Science, Medicine & Health, University of Wollongong, Sydney, NSW Australia; 4 Caring Futures Institute, College of Nursing and Health Science, Flinders University, Adelaide, SA Australia; 5 McGrath Foundation, Sydney, NSW Australia; 6 Human Performance Research Centre, INSIGHT Research Institute, University of Technology Sydney (UTS), Sydney, NSW Australia; 7 Exercise Medicine Research Institute, School of Medical and Health Sciences, Edith Cowan University, Perth, WA Australia; 8 Institute for Health Research, University of Notre Dame Australia, Perth, WA Australia; 9 Department of Nursing, School of Health Sciences, University of Melbourne, Melbourne, VIC Australia; 10 Department of Health Services Research, Peter MacCallum Cancer Centre, Melbourne, Australia; 11 Sir Peter MacCallum Department of Oncology, University of Melbourne, Parkville, VIC Australia; 12 Victorian Comprehensive Cancer Centre Alliance, Melbourne, VIC Australia; 13 Flinders Health and Medical Research Institute, College of Medicine and Public Health, Flinders University, Adelaide, SA Australia; 14 Flinders Medical Centre, Adelaide, SA Australia; 15 Oncology-Haematology (Paediatric), Royal Hobart Hospital, Hobart, TAS Australia; 16 Division of Paediatrics, Medical School, University of Western Australia, Perth, WA Australia; 17 Department of Medicine, School of Translational Medicine, Monash University, Melbourne, VIC Australia; 18 Department of Medical Oncology, Alfred Health, Melbourne, VIC Australia; 19 Nutrition and Speech Pathology Department, Peter MacCallum Cancer Centre, Melbourne, VIC Australia; 20 Healthability, Box Hill, VIC, Australia; 21 Department of Health Services Research, Peter MacCallum Cancer Centre, Melbourne, VIC Australia; 22 Australian Cancer Survivorship Centre, Peter MacCallum Cancer Centre, Melbourne, VIC Australia

**Keywords:** Cancer survivorship, Delphi study, Nurse capabilities, Nurse-led care

## Abstract

**Purpose:**

To establish capabilities required by nurses to deliver quality cancer survivorship care in Australia.

**Methods:**

A two-round online modified Delphi involving Australian cancer nurses. Initial domains and capability statements were based on the Quality of Cancer Survivorship Care Framework and supplemented by national and international nursing frameworks. In Round 1 (R1), experts categorised the applicability of 53 capabilities for cancer nurses, across eight domains, in relation to Australian National Professional Development Framework for Cancer Nursing (EdCaN) groups: ‘All’, ‘Many’, ‘Some’, and ‘Few’ nurses, or not relevant. In Round 2 (R2), experts rated agreement with capabilities allocated to the nurse groups. A priori consensus was set at ≥ 80%.

**Results:**

Surveys were distributed to 51 experts, with a response rate of 92% (47/51) for R1 and 75% (38/51) for R2. Following R1, ten capabilities were added, resulting in 63 capabilities for R2 to establish consensus allocation to EdCaN groupings. Fifty-seven capabilities reached consensus; four capabilities were moved from ‘many’ to ‘some’ nurses; one capability was moved from ‘some’ to ‘few’ nurses; and one capability was retained in ‘all’ nurses following Delphi feedback and research team discussion.

**Conclusions:**

Sixty-three capabilities across eight cancer survivorship care domains were identified and allocated to different nursing groupings. This study provides important foundational work by identifying the capabilities of cancer nurses to deliver quality cancer survivorship care in Australia.

**Implications for Cancer Survivors:**

The identification of clearly defined capabilities may improve the quality of cancer survivorship care through the enrichment and standardisation of educational curricula and continuing professional education, and through improved workforce planning.

**Supplementary information:**

The online version contains supplementary material available at https://doi.org/10.1007/s11764-025-01804-6.

## Introduction

Cancer is a significant global health issue, affecting millions worldwide and contributing substantially to mortality rates and healthcare expenditure. The International Agency for Research on Cancer (IARC) estimated 20 million new cases and almost 10 million deaths in 2022, with predictions of 35 million new diagnoses annually by 2025 [1]. In Australia, cancer accounts for 17% of the ill health burden, 32% of deaths, and 10% of health system expenditure [2, 3]. Despite these challenges, the 5-year cancer survival rate in Australia and other developed economies has improved by almost 20% over the past 30 years, now exceeding 70% for all cancers [4]. As a result, the number of cancer survivors is increasing worldwide, with the latest reports estimating over 18.1 million survivors in the United States, almost 24 million in Europe and 1.2 million in Australia [4–7]. However, life after a cancer diagnosis presents significant ongoing challenges affecting every body system, with cancer survivors at risk of relapse, secondary cancer, and comorbid chronic illness as well as a range of emotional and psychosocial issues [8–13]. Consequently, survivors often have difficulty resuming previous roles, such as returning to work or reintegrating into school [9, 12, 14, 15]. These issues are persistent, are under-recognised, and result in substantial unmet needs among cancer survivors [14, 16, 17]. This underscores the importance of preparing and supporting people for life after a cancer diagnosis [18]—a notion that gained international recognition 20 years ago in the 2005 Institute of Medicine (IOM) report, ‘From Cancer Patient to Cancer Survivor: Lost in Transition’ [9].

Despite the IOM recommendations, the provision of cancer survivorship care is highly variable across Australia, and internationally, with few survivors having access to comprehensive and well-funded dedicated services [19–22]. Cancer nurses, as the largest cancer care workforce, are uniquely positioned to provide ongoing, person-centred survivorship care due to their expertise in helping patients manage the physical, emotional, and practical challenges of cancer, and their ability to navigate Australia’s complex, multi-level healthcare system [20, 23, 24]. Additionally, cancer nurses possess valuable skills in providing self-management support, health promotion advice and counselling, and appropriate referrals to services [20, 23, 24]. The integration of survivorship care into oncology nursing practice is crucial for addressing the complex needs of cancer survivors and enhancing their overall well-being. Given the growing numbers of survivors and the well-established limits of oncologist-led care [9, 22], there is an urgent need to ensure consistent access to quality survivorship care and to define the capabilities of the cancer nursing workforce to inform educational curricula and practice frameworks for all nurses who care for people affected by cancer.

The aim of this study was to establish expert consensus on capabilities required by cancer nurses in Australia to deliver quality cancer survivorship care. In the context of this study, cancer survivorship care was defined as care provided during the post-treatment period, recognising it may include those receiving long-term therapies, while explicitly excluding end-of-life care. Defining these capabilities, such as skills, knowledge, values, and confidence [25], is essential for informing curriculum development, enhancing professional training, and influencing policy changes related to healthcare delivery and support.

## Methods

### Study design

A two-round modified Delphi approach was used to achieve consensus by gathering structured and practical feedback with a select group of experts [26, 27]. The first round (Round 1; R1) aimed to identify capability statements relevant to cancer nurses and asked experts to categorise each statement according to different nurse groups as defined by the Australian National Professional Development Framework for Cancer Nursing (EdCaN) [28]. The EdCaN framework was chosen as it represents a national professional development framework designed to enhance the capabilities of cancer nurses by providing structured pathways for learning and specialisation, ensuring that nurses can meet core competencies in cancer control and advance in their careers. EdCaN defines nurse capabilities across levels of clinical competency as applicable to ‘All’, ‘Many’, ‘Some’, or ‘Few’ nurses (Supplementary file 1). ‘All’ refers to nurses able to ‘demonstrate national competency standards for registered nurses as described by the Australian Nursing & Midwifery Council but applied to cancer control’. ‘Many’ nurses should have the same ability as ‘All’ nurses but ‘at a more advanced level’. ‘Some’ nurses refer to those practising at specialist cancer nursing standards. ‘Few’ nurses represent those ‘credentialed to practise at an advanced level or in extended practice roles’ [28]. The second Delphi round (Round 2; R2) aimed to determine the level of agreement with the capabilities assigned to each nurse group as indicated after R1. In both rounds, participants were advised of the purpose of the study, given specific instructions for the survey, and provided with the definition of the EdCaN professional framework nurse groups (Supplementary file 1). The surveys were administered through REDCap [29].

### Development of capability statements

Initial domains and capability statements for nurses to provide quality cancer survivorship care in Australia were derived from the Quality of Cancer Survivorship Care Framework (QCSCF) [30]. These were further refined through discussions with the research team, who are all members of the Clinical Oncology Society of Australia (COSA) Survivorship Group, and multi-professional experts in cancer nursing, cancer survivorship care and research. The QCSCF was assessed as the most relevant framework for the aim of this study, as it outlines the domains and factors essential for delivering quality, comprehensive, evidence-based cancer survivorship care.

Proposed capabilities within the QCSCF were adapted into 53 items across eight domains, which comprehensively represent nurse-relevant capabilities for cancer survivorship care. To ensure rigour and that no nursing specific capabilities were omitted from the item list, the principal investigator (GM) reviewed the capability statements against existing cancer nurse competency frameworks, including the 2009 Australian based EdCaN framework [28], the European Cancer Nursing Education Framework (EONS) [31], the Canadian, Nursing Knowledge and Practice Framework and Toolkit for Cancer Care (CANO) Framework [32], and the NHS Career Pathways Core Cancer Capabilities and Education Framework [33]. It was determined that no additional capabilities were deemed necessary for the generated item list, as all relevant domains and capabilities were represented.

Pilot testing of R1 was conducted with the research team and four cancer nurses external to the research team with significant experience in the care of cancer survivors. Participants of the pilot testing provided feedback on the survey’s usability, content, and length. No changes were required following testing.

### Expert panel selection

Potential participants in the Delphi study were nurses working in specialised roles related to cancer survivorship, including cancer nurse educators, nursing working in inpatient and outpatient cancer settings, cancer nurse researchers, and nurses in non-cancer settings such as general practice and genomics. Eligibility criteria included having at least five years of experience in cancer nursing, residing and working in Australia, being 18 years or older, and being proficient in English.

We planned to recruit a minimum of 30 experts. The recruitment strategy sought to include at least one representative from each state and territory, as well as from a quaternary cancer centre, a tertiary cancer centre, a regional cancer centre and primary care. Additionally, the panel sought to include nurses who care for cancer survivors across all life stages, including paediatrics, adolescents and young adults (AYA), adults, and geriatrics. Experts were purposefully selected by the research team through known networks, including COSA, Cancer Nursing Society of Australia (CNSA), and the Australian and New Zealand Children’s Haematology/Oncology Group (ANZCHOG) memberships, along with the study researchers’ professional connections.

Nurses willing to participate in the Delphi study were provided with an information sheet detailing the purpose of the study, participation requirements, and eligibility criteria. Participants were given three weeks to respond to each survey round. An invitation to attend a consensus meeting was planned if consensus was not reached after R2. In instances of non-response for either round, up to two email reminders were sent. All experts were invited to R2 regardless of their participation in R1.

### Round 1 procedure

Experts were asked to provide demographic details including age, gender, role, work location, expert group, years of experience in nursing, and years of experience in cancer survivorship care. Experts were then presented with 53 capability statements across eight domains. For each statement, experts were asked to assess its relevance for nurses in delivering quality cancer survivorship care and then categorise each capability to the relevant EdCaN professional development framework nurse group [28] (e.g. 1 = All, 2 = Many, 3 = Some, 4 = Few, and 5 = Not Relevant). Additionally, participants were invited to provide comments on each capability statement and to suggest any capabilities they believed were missing from the list.

### Round 2 procedure

The revised capability statements from R1 were presented to the experts according to nurse group (‘All’, ‘Many’, ‘Some’, and ‘Few’). Experts were asked to rate their level of agreement with the nursing groups assigned to each capability statement using a 5-point Likert scale (1 = Strongly Disagree, 2 = Disagree, 3 = Neutral, 4 = Agree, and 5 = Strongly Agree). If experts did not agree with the assigned group (Likert score 1–3), they were asked to suggest an alternate nurse group for the item and provide a rationale for their proposed reassignment.

### Data analysis

Quantitative data were analysed and summarised using descriptive statistics. Frequencies, percentages, medians, and ranges were calculated using IBM Statistical Package for the Social Sciences (SPSS©, v28.0). A content analysis was performed on qualitative, free-text responses.

A priori thresholds for agreement were adapted from previous cancer research Delphi studies [26, 34], with nurse capability statements retained in a specific nurse group if 80% or more of the experts agreed on the assignment (Likert score 4–5). In R1, statements that received 60% to 79% agreement were subject to review by the research team. Conversely, items deemed not applicable to nurses by 60% or more of the experts were excluded from consideration in R2. The research team considered qualitative feedback from experts when reviewing statements with 60% to 79% agreement and when rewording existing capability statements. In R2, items that received less than 80% agreement were reviewed by the research team, who considered the qualitative feedback provided by experts, and deliberated via written correspondence (email) to reach an agreement on whether the capability statement should be retained in the assigned nurse group or reallocated to a more relevant group.

## Results

Invitations to participate in each survey round were sent to 51 experts. For R1, there were 47 participants who completed the survey (92% response rate) and in R2, there were 38 participants who completed the survey (75% response rate). Demographic characteristics of the sample for each survey is presented in Table 1. All Australian states were represented in both surveys, while no experts were recruited from the two Australian territories. More than 90% of the respondents in each survey were female; the majority were aged 40 to 49 years and were located in Victoria, New South Wales, and Queensland (Australia’s three most populous states). Quaternary, tertiary, and regional cancer centres were the most common work setting. Less than 11% of the participants in each round identified the university, specialised non-cancer service, or other (not-for-profit charity) as their work setting. No representative from specialised non-cancer services completed R2 survey.

**Table 1:** Expert panel demographics

Demographics	Round 1 *n*= 47 *N*(%)	Round 2 *n*= 38 *N*(%)
**Age range** (years)		
30–39	10 (21.3%)	8 (21.1%)
40–49	19 (40.4%)	17 (44.7%)
50–59	12 (25.5%)	7 (18.4%)
60+	6 (12.8%)	6 (15.8%)
**Gender**		
Female	44(93.6%)	35 (92.1%)
Male	3(6.4%)	3(7.9%)
**Location** (states)		
New South Wales	14 (29.8%)	11 (28.9%)
Queensland	8 (17.0%)	6 (15.8%)
South Australia	3 (6.4%)	3 (7.9%)
Tasmania	2 (4.3%)	2 (5.3%)
Victoria	16 (34.0%)	12 (31.6%)
Western Australia	4 (8.5%)	4 (10.5%)
**Work setting**		
Quaternary/tertiary cancer centre	33 (70.2%)	29 (76.3%)
Regional cancer centre	9 (19.1%)	5 (13.2%)
University	3 (6.4%)	3 (7.9%)
Specialised non-cancer service relevant to cancer care	1 (2.1%)	0 (0%)
Other: Not-for-profit charity	1 (2.1%)	1 (2.6%)
**Area (if working in hospital setting)**		
Hospital outpatient clinics	23 (60.5%)	21 (70%)
Hospital inpatient care	4 (10.5%)	3 (10%)
Oncology day treatment centre	8 (21.2%)	3 (10%)
Other (Community, Primary Health, Research, GP liaison)	3 (7.9%)	3 (10%)
**Main role**		
Clinician nurse consultant	20 (42.6%)	17 (44.7%)
Nurse practitioner/nurse practitioner candidate	9 (19.1%)	9 (23.7%)
Clinical nurse specialist/clinical nurse	6 (12.8%)	2 (5.3%)
Nurse researcher/academic	5 (10.6%)	6 (15.8%)
Clinical nurse educator	2 (4.3%)	0 (0%)
Registered nurse	2 (4.3%)	1 (2.6%)
Nurse manager/nurse unit manager	2 (4.3%)	2 (5.3%)
Nurse Coordinator	1 (2.1%)	1 (2.6%)
**Patient population** (age group) (could tick > 1)		
Adults	40 (58.8%)	30 (60%)
Geriatrics	14 (20.6%)	10 (20%)
Adolescents and young adults	11 (16.2%)	7 (14%)
Paediatrics	3 (4.4%)	3 (6%)
**Years of experience in nursing**	Mean 25.3 years Range 7–46	Mean 24.6 years Range 7–46
Years of experience in cancer nursing	Mean 20.5 years Range 4–37	Mean 20.5 years Range 6–37
Area of expertise (could tick > 1)		
General cancer care (clinical)	27 (38.6%)	23 (40.4%)
Cancer survivorship care	20 (28.6%)	15 (26.3%)
Cancer nursing education	13 (18.6)	9 (15.8%)
Cancer nursing research	8 (11.4%)	9 (15.8%)
Specialised non-care role relevant to cancer care	1 (1.4%)	0 (0%)
General practice	1 (1.4%)	1 (1.8%)
**Years of experience in cancer survivorship care**	Mean 8.5 years Range 3–15	Mean 8.6 years Range 1–15
**Highest level of Education**		
Masters	22 (46.8%)	21 (55.3%)
Postgrad certificate	13 (27.7%)	8 (21.1%)
Postgrad diploma	5 (10.6%)	2 (5.3%)
PhD	4 (8.5%)	4 (10.4%)
Bachelor of Nursing	2 (4.3%)	2 (5.3%)
Other (undertaking Masters)	1 (2.1%)	1 (2.6%)

### Round 1

A total of 53 capability statements were rated in R1. The domain titles and number of capability statements presented in Round 1 were as follows: (1) prevention and surveillance for recurrence and new cancers (*n* = 8), (2) surveillance and management of physical effects (*n* = 8), (3) surveillance and management of psychosocial effects (*n* = 9), (4) surveillance and management of chronic medical conditions (*n* = 2), (5) health promotion and disease prevention (*n* = 7), (6) communication/decision making (*n* = 9), (7) care coordination (*n* = 5), and (8) cancer survivor/carer experience (*n* = 5).

No capability statement achieved more than 80% agreement from the expert panel, nor were any deemed irrelevant by 60% or more of the experts. As a result, all capability items underwent a review by the research team, who also considered the qualitative feedback provided by the experts. This review process was led by cancer nursing experts in the research team (GM, RJC, and MK) who facilitated discussions on assigning capabilities to nurse groups. These discussions occurred through verbal exchanges (ZOOM meetings) and written correspondence (email). Based on these deliberations, capability items were assigned to nurse groups that received the highest ratings from experts. Often, results clustered around a single category; for instance, the largest percentage of experts might have assigned a capability item to ‘Some’ nurses, with smaller percentages allocating the item to ‘Many’ or ‘Few’. Free text feedback including suggestions of the preferred nurse groups were also taken into consideration by the research team, enabling consensus to be visible among the results by the research team.

Based on the qualitative feedback, the research team refined several capability statements that originally combined multiple actions, resulting in the addition of 10 new statements in R2. For example, “makes referrals for and/or coordinates recommended evaluations including, as indicated, laboratory, imaging, and/or specialty care for physical health” was separated into (i) makes referrals for recommended evaluations including, as indicated, laboratory, imaging, and/or specialty care for physical health and (ii) coordinates recommended evaluations including, as indicated, laboratory, imaging, and/or specialty care for physical health. These revisions were made to enhance clarity and ensure that the items accurately reflected their intended capability.

### Round 2

A total of 63 capability statements were rated in R2. Compared to R1, the number of capability statements increased in four domains. Specifically, prevention and surveillance for recurrence and new cancers increased by three to 11 statements, surveillance and management of physical effects increased by two to 10 statements, surveillance and management of psychosocial effects increased by one to 10 statements, and health promotion and disease prevention increased by four to 11 statements. In contrast, the domains surveillance and management of chronic medical conditions, communication/decision making, care coordination, and cancer survivor/carer experience remained unchanged from R1.

Fifty-seven statements achieved more than 80% agreement (Table 2). The six items that received scores below this threshold, and the item’s respective qualitative feedback, were reviewed by the research team. Researchers determined four of the capability statements should be reassigned from ‘Many’ to ‘Some’ nurses, one capability statement was reassigned from ‘Some’ to ‘Few’ nurses, and one capability statement was retained in ‘All’ nurses.

**Table 2:** Round 2 Delphi results—expert panel agreement (%) with allocated nurse group

Domain	Capability statement	%
Agreement with **ALL** nurse group		
Surveillance and management of physical effects	Assess frailty	97.4
	Assess overall burden of physical symptoms	92.1
	Provide recommendations for risk-reducing strategies (e.g. weight loss and exercise)	94.8
Surveillance and management of psychosocial effects	Assess psychosocial symptoms and/or conditions using history or validated instruments, including those that are general and tailored by cancer type and/or treatment exposure. (e.g. fatigue, anxiety, distress, fear of recurrence, and sleep disturbance)	86.8
	Assess financial and employment issues/challenges (e.g. financial toxicity, underemployment, unemployment, return-to-work, and work/school productivity)	76.3
	Assess age-appropriate interpersonal issues (e.g. sexuality, fertility (including fertility preservation) family/caregiver, and friend/social relationships)	81.5
	Make referrals for recommended investigations/evaluations (e.g. psychosocial screening and referral to specialty care for psychosocial assessment)	86.8
Health promotion and disease prevention	Assess lifestyle behaviours, and coordinate referrals/treatment (e.g. smoking, alcohol, and sun protection)	97.7
Communication/decision making	Provide holistic care and include family members or friends in discussions	100
	Advocate and enable shared decision making (e.g. by assessing risk perception, values, decision support, and what matters most to people)	100
	Maintain respectful communication with cancer survivors	100
	Provide care consistent with cancer survivors’ goals of care	100
Care coordination	Communicate with cancer survivors and carer/guardian/family members all relevant cancer survivorship care information	86.8
Cancer survivor/carer experience	Evaluate cancer survivors’ satisfaction with their healthcare provider and/or the healthcare delivery setting	92.1
	Evaluate carer/guardian/family members’ satisfaction with their cancer survivor’s healthcare provider and/or healthcare setting	92.1
	Evaluate the quality and safety of care provided to cancer survivors	92.1
	Evaluate the degree to which the cancer survivor was involved as an active participant in their own care and the adequacy of the communication with them and the education provided to them	92
Agreement with **MANY** nurse group		
Surveillance and management of physical effects	Assess physical symptoms and/or conditions via history, physical examination, and/or standardised instruments, tailored by cancer type, treatment exposure and age when treated	92.1
	Assess exercise tolerance as per scope of practice	84.2
	Coordinate recommended investigations/evaluations including, as indicated, laboratory, imaging, and/or specialty care for physical health as per cancer survivor condition	65.8
	Coordinate recommended treatment, such as medication, therapy, and/or exercise as per cancer survivor condition	71.1
	Make referrals for recommended investigations/evaluations including, as indicated, laboratory, imaging, and/or specialty care for physical health	55.3
	Make referrals for recommended treatment, such as medication, therapy, and/or exercise as per cancer survivor condition	57.9
	Coordinate recommended investigations/evaluations (e.g. psychosocial screening and referral to specialty care for psychosocial assessment)	94.7
	Assess benefit and harms of treatment provided for psychological health (e.g. medication, therapy, and exercise)	86.8
Health promotion and disease prevention	Coordinate referral/treatment for weight management (e.g. obesity, physical activity, and diet)	89.5
	Assess weight management (e.g. obesity, physical activity, and diet)	86.8
Communication/decision making	Educate and assess cancer survivors’ understanding of their illness and care (including health literacy)	100
	Assess cancer survivors’ self-management capability and provide advice/education	92.1
	Discuss sensitive topics (e.g. sexual activity, continence, and end-of-life care)	84.2
Agreement with **SOME** nurse group		
Prevention and surveillance for recurrence and new cancers	Provide recommendations for adjuvant and/or risk reducing strategies	94.7
	Coordinate clinical surveillance reviews as recommended in relevant guidelines	89.5
	Coordinate laboratory surveillance testing as recommended in relevant guidelines	86.8
	Coordinate imaging surveillance as recommended in relevant guidelines	86.8
	Assess adherence with recommended adjuvant and/or risk reducing strategies	89.5
	Perform clinical surveillance reviews as recommended in relevant guidelines	92.1
	Review reports of requested investigations/evaluations (e.g. genetic, laboratory and imaging reports) and action as per scope of practice	84.2
Surveillance and management of physical effects	Re-assess physical symptoms and/or conditions at defined intervals and/or treatment phases as per cancer survivor situation	94.8
	Assess barriers to education, if attending school, as a consequence of cancer survivor’s illness (e.g. development and learning, attendance, access, and friendship networks)	92.1
	Review reports of requested investigations/evaluations pertaining to psychosocial care and action appropriately (e.g. make referral to speciality care or for further testing/imaging)	92.1
	Assess adherence to treatment for psychological health	92.1
	Re-assess psychosocial symptoms and/or conditions at defined intervals and/or treatment phases	86.9
Surveillance and management of chronic medical conditions	Evaluate and make age-specific recommendations for treatment of non-cancer medical conditions (e.g. growth and development, adolescent health, hypertension, diabetes, and depression) using disease- and age-specific indicators as relevant to the cancer survivor	84.3
Health promotion and disease prevention	Coordinate prevention-focused reviews and testing (e.g. screening for diabetes, hypertension, and hyperlipidaemia)	84.2
	Coordinate age- and gender-appropriate cancer screening (e.g. pap smear, mammogram, and colonoscopy)	86.8
	Perform prevention-focused reviews and testing (e.g. screening for diabetes, hypertension, and hyperlipidaemia)	84.2
	Perform age- and gender-appropriate cancer screening (e.g. pap smear, mammogram, and colonoscopy)	71
	Provide vaccination advice and assess vaccination rates (e.g. influenza, pneumonia, meningococcal, and shingles, particularly among those who may be chronically immunocompromised)	92.1
	Administer vaccinations (e.g. influenza, pneumonia, meningococcal, and shingles, particularly among those who may be chronically immunocompromised)	84.2
	Screen for exposure to infectious diseases (e.g. HIV, hepatitis b, and hepatitis c)	92.1
Communication/decision making	Develop cancer survivorship plans for each cancer survivor	94.7
	Discuss and document advance care planning	89.5
Care coordination	Document care plans and share with cancer survivors’ care team (e.g. provision of tailored survivorship care plans)	97.3
	Educate other healthcare professionals involved in the cancer survivors’ care about their medical history and ongoing care needs	97.4
	Communicate with oncology specialists, primary care providers and other healthcare professionals	97.4
Cancer survivor/carer experience	Evaluate the timeliness of cancer survivors’ access to relevant care services	92.1
Agreement with **FEW** nurse group		
Prevention and surveillance for recurrence and new cancers	Assess risk predisposition including family history, for recurrence and new cancer	92.1
	Make referrals for genetic testing as recommended in relevant guidelines	92.1
	Order laboratory surveillance testing as recommended in relevant guidelines	89.5
	Order imaging surveillance as recommended in relevant guidelines	90.4
Surveillance and management of chronic medical conditions	Perform medication reconciliation	81.6
	Review reports of requested screening investigations (Pap smears, etc.) and action appropriately	81.5
Care coordination	Assess risk of polypharmacy and important medication interactions	86.8

Among the 63 capability items in R2, two pertained to making referrals, which each had the lowest agreement rates across all capability items, falling between 50 and 60%. Specifically, the capability item “many nurses should have the capability to make referrals for recommended investigations/evaluations including, as indicated, laboratory, imaging, and/or specialty care for physical health” achieved 55.3% agreement. Similarly, the item “many nurses should have the capability to make referrals for recommended treatment, such as medication, therapy, and/or exercise as per cancer survivor condition” reached 57.9% agreement. The expert panel’s aggregated qualitative feedback for these two items consistently referenced the need for specialised or advanced training and association with the Nurse Practitioner role, thus the research team rationalised the reassignment of these two items to the ‘Some’ nurse group. It is important to note that according to the EdCaN definition, ‘Few’ relates primarily to nurse practitioners (Supplementary file 1).

The nurse group and number of capabilities that reached consensus in R2 were as follows: In the ‘All’ nurse group, 16 out of 17 capabilities reached consensus. In the ‘Many’ nurse group, 9 out of 13 capabilities reached consensus, with two capabilities that did not reach consensus being from Domain 2: surveillance and management of physical effects. In the ‘Some’ nurse group, 26 out of 27 capabilities reached consensus. Finally, in the ‘Few’ nurse group, six out of six capabilities reached consensus. The final list of capabilities for nurses to provide quality cancer survivorship care in Australia, by the nurse group, can be found in Table 3.

**Table 3:** Capabilities for nurses to provide quality cancer survivorship care in Australia by nurse group

Domain	Capability	All	Many	Some	Few
Prevention and surveillance for recurrence and new cancers	1. Provide recommendations for adjuvant and/or risk reducing strategies			x	
	2. Coordinate clinical surveillance reviews as recommended in relevant guidelines			x	
	3. Coordinate laboratory surveillance testing as recommended in relevant guidelines			x	
	4. Coordinate imaging surveillance as recommended in relevant guidelines			x	
	5. Assess adherence with recommended adjuvant and/or risk-reducing strategies			x	
	6. Perform clinical surveillance reviews as recommended in relevant guidelines			x	
	7. Review reports of requested investigations/evaluations (e.g. genetic, laboratory and imaging reports) and action as per scope of practice			x	
	8. Assess risk predisposition including family history, for recurrence and new cancer				x
	9. Make referrals for genetic testing as recommended in relevant guidelines				x
	10. Order laboratory surveillance testing as recommended in relevant guidelines				x
	11. Order imaging surveillance as recommended in relevant guidelines				x
Surveillance and management of physical effects	12. Assess frailty	x			
	13. Assess overall burden of physical symptoms	x			
	14. Provide recommendations for risk-reducing strategies (e.g. weight loss and exercise)	x			
	15. Assess physical symptoms and/or conditions via history, physical examination, and/or standardised instruments, tailored by cancer type, treatment exposure and age when treated		x		
	16. Assess exercise tolerance as per scope of practice		x		
	17. Coordinate recommended investigations/evaluations including, as indicated, laboratory, imaging, and/or specialty care for physical health as per cancer survivor condition			x	
	18. Coordinate recommended treatment, such as medication, therapy, and/or exercise as per cancer survivor condition			x	
	19. Make referrals for recommended investigations/evaluations including, as indicated, laboratory, imaging, and/or specialty care for physical health			x	
	20. Make referrals for recommended treatment, such as medication, therapy, and/or exercise as per cancer survivor condition			x	
	21. Re-assess physical symptoms and/or conditions at defined intervals and/or treatment phases as per cancer survivor situation			x	
Surveillance and management of psychosocial effects	22. Assess psychosocial symptoms and/or conditions using history or validated instruments, including those that are general and tailored by cancer type and/or treatment exposure. (e.g. fatigue, anxiety, distress, fear of recurrence, and sleep disturbance)	x			
	23. Assess financial and employment issues/challenges (e.g. financial toxicity, underemployment, unemployment, return-to-work, work/school productivity)	x			
	24. Assess age-appropriate interpersonal issues (e.g. sexuality, fertility (including fertility preservation), family/caregiver and friend/social relationships)	x			
	25. Make referrals for recommended investigations/evaluations (e.g. psychosocial screening and referral to specialty care for psychosocial assessment)	x			
	26. Coordinate recommended investigations/evaluations (e.g. psychosocial screening and referral to specialty care for psychosocial assessment)		x		
	27. Assess benefit and harms of treatment provided for psychological health (e.g. medication, therapy, and exercise)		x		
	28. Assess barriers to education, if attending school, as a consequence of cancer survivor’s illness (e.g. development and learning, attendance, access, and friendship networks)			x	
	29. Review reports of requested investigations/evaluations pertaining to psychosocial care and action appropriately (e.g. make referral to speciality care or for further testing/imaging)			x	
	30. Assess adherence to treatment for psychological health			x	
	31. Re-assess psychosocial symptoms and/or conditions at defined intervals and/or treatment phases			x	
Surveillance and management of chronic medical conditions	32. Evaluate and make age-specific recommendations for treatment of non-cancer medical conditions (e.g. growth and development, adolescent health, hypertension, diabetes, and depression) using disease- and age-specific indicators as relevant to the cancer survivor			x	
	33. Perform medication reconciliation				x
Health promotion and disease prevention	34. Assess lifestyle behaviours, and coordinate referrals/treatment (e.g. smoking, alcohol, and sun protection)	x			
	35. Coordinate referral/treatment for weight management (e.g. obesity, physical activity, and diet)		x		
	36. Assess weight management (e.g. obesity, physical activity, diet)		x		
	37. Coordinate prevention-focused reviews and testing (e.g. screening for diabetes, hypertension, and hyperlipidaemia)			x	
	38. Coordinate age- and gender-appropriate cancer screening (e.g. pap smear, mammogram, colonoscopy)			x	
	39. Perform prevention-focused reviews and testing (e.g. screening for diabetes, hypertension, hyperlipidaemia)			x	
	40. Perform age- and gender-appropriate cancer screening (e.g. pap smear, mammogram, and colonoscopy)				x
	41. Provide vaccination advice and assess vaccination rates (e.g. influenza, pneumonia, meningococcal, shingles, particularly among those who may be chronically immunocompromised)			x	
	42. Administer vaccinations (e.g. influenza, pneumonia, meningococcal, and shingles, particularly among those who may be chronically immunocompromised)			x	
	43. Screen for exposure to infectious diseases (e.g. HIV, hepatitis b, and hepatitis c)			x	
	44. Review reports of requested screening investigations (Pap smears, etc.) and action appropriately			x	
Communication/decision making	45. Provide holistic care and include family members or friends in discussions	x			
	46. Advocate and enable shared decision making (e.g. by assessing risk perception, values, decision support and what matters most to people)	x			
	47. Maintain respectful communication with cancer survivors	x			
	48. Provide care consistent with cancer survivors’ goals of care	x			
	49. Educate and assess cancer survivors’ understanding of their illness and care (including health literacy)		x		
	50. Assess cancer survivors’ self-management capability and provide advice/education		x		
	51. Discuss sensitive topics (e.g. sexual activity, continence, and end-of-life care)		x		
	52. Develop cancer survivorship plans for each cancer survivor			x	
	53. Discuss and document advance care planning			x	
Care coordination	54. Communicate with cancer survivors and carer/guardian/family members all relevant cancer survivorship care information	x			
	55. Document care plans and share with cancer survivors’ care team (e.g. provision of tailored survivorship care plans)			x	
	56. Educate other healthcare professionals involved in the cancer survivors’ care about their medical history and ongoing care needs			x	
	57. Communicate with oncology specialists, primary care providers and other healthcare professionals			x	
	58. Assess risk of polypharmacy and important medication interactions				x
Cancer survivor/carer experience	59. Evaluate cancer survivors’ satisfaction with their healthcare provider and/or the healthcare delivery setting	x			
	60. Evaluate carer/guardian/family members’ satisfaction with their cancer survivor’s healthcare provider and/or healthcare setting	x			
	61. Evaluate the quality and safety of care provided to cancer survivors	x			
	62. Evaluate the degree to which the cancer survivor was involved as an active participant in their own care and the adequacy of the communication with them and the education provided to them	x			
	63. Evaluate the timeliness of cancer survivors’ access to relevant care services			x	

## Discussion

This study identified essential capabilities for the delivery of quality cancer survivorship care by Australian cancer nurses, from novice to expert practitioners. Findings from the study align with international recommendations for the delivery of high-quality cancer care [35]. While many of the identified capabilities may also be applicable to other professions, nurses possess a combination of skills and knowledge that position them to make distinct contributions to cancer survivorship care. These include knowledge and understanding of cancer and cancer treatments, their proximity to patients which enables the delivery and undertaking of comprehensive holistic assessments, symptom management and patient education, care coordination across specialties and settings, and the provision of psychosocial support.

While our study defines the capabilities of cancer care nurses to deliver quality cancer survivorship care, the ability to practise these capabilities depends on factors including workforce and funding availability, infrastructure, funders willingness, and the culture and politics of healthcare. The projected nursing shortfall of approximately 80,000 nurses in Australia by 2035 suggests that simply defining the capabilities required of cancer nurses will *not* be sufficient to ensure quality survivorship care [36]. Difficult decisions may need to be made to prioritise more intensive nursing survivorship care, such as for survivors with complex needs, for underserved communities, survivors with limited health literacy, and young adult survivors.

The capability framework should also be applied in a way that accommodates the diverse characteristics of the nursing workforce across Australia, particularly in rural and remote regions. In these areas, patients often lack access to comprehensive cancer services and are more likely to receive healthcare from nurses who are not oncology focused, such as primary care nurses [4, 7]. It is expected that these nurses could still provide quality cancer survivorship care according to the capabilities for ‘All’ nurses in the framework, but may need additional training and support. Increased resources to adequately staff and support nurse-led care for cancer survivors should be prioritised by service planners, as nurse-led survivorship models may be associated with increased patient and caregiver satisfaction, cost-effectiveness, and comparable outcomes in emergency service utilisation and hospitalisations when compared to specialist-led care [37]. Political, regulatory, institutional and cultural change will be needed to overcome resistance to changing from traditional specialist-led care to enable cancer nurses to fully develop and execute the identified capabilities and to ensure equitable access to quality cancer survivorship care [38].

The findings of this study provide valuable data for employers to refine role descriptions, funding organisations to allocate resources for the cancer nursing workforce, and education providers to determine the clinical skills and educational needs of cancer nurses. These results may also be relevant to other countries with similar health systems and nurse-led cancer survivorship care models, aligning with existing frameworks in the UK, Europe, Canada, and Australia [28, 31–33].

While the results of this study provide insight into the delivery of cancer survivorship care, broader issues related to the health infrastructure are beyond the scope of this study. Specifically, the subdomain of Clinical Structure within the Health-Care Delivery domain from QCSCF was excluded during the Delphi process. This subdomain includes availability of specialty care and relevant health professionals in the provision of holistic care, use of health information systems, accessibility of care for patients, and opportunities for research participation by cancer survivors [30]. As a consequence, this Delphi study does not provide insights into cancer nurses’ perspective of availability of and access to health infrastructure or services which may impact their ability to provide cancer survivorship care and would be valuable to understand in influencing future policy, research and development in this area.

### Strengths and limitations

This study had a number of strengths and limitations. We undertook an online Delphi survey which increased the timeliness of deploying surveys and receiving responses from participants by increasing accessibility. It also allowed researchers to include expert panellists located throughout Australia. All states were represented in both rounds, and participants with a range of ages, work setting and area, main roles, patient population experience, area of expertise, years of experience and education were included. The response rates were high compared to other similar studies, and the demographic characteristics were similar in both rounds. This ensured dependability through consistent characteristics of the cancer survivorship nursing workforce across both surveys. Confirmability was addressed by the anonymised questionnaire, analysis of numeric data and aggregation of qualitative responses from panellists helped minimise bias and maintain impartiality from researchers.

However, we acknowledge several limitations. Firstly, there was a metropolitan bias in participation, with only 19% and 13% of participants from regional cancer centres in R1 and R2 respectively. Additionally, we recognise the lack of non-specialist representation on the panel, which may limit the generalisability of our findings to broader populations.

Furthermore, not all panellists provided a mandatory rationale, as instructed, for responses where they were not in agreement (‘Strongly disagree’, ‘Disagree’ or ‘Neutral’) with the capability items and its assigned nurse groups. The aggregated qualitative feedback was limited to rationales that were provided. While panellists were purposefully selected by the research team through known professional networks, enabling a wide representation from relevant experts, it is still possible that responses reflect the dominance of particular groups, and may not be fully representative.

## Conclusion

In conclusion, this study has successfully developed a comprehensive list of capabilities for nurses to deliver cancer survivorship care in Australia using a modified Delphi technique. As the first study of its kind in Australia, this work establishes a foundation for future research on the cancer nursing workforce and has the potential to inform subsequent research on career development, educational curriculum, research opportunities, and resources needed for nurses and other healthcare professionals involved in cancer survivorship care [39]. By identifying nurses’ key roles and capabilities in the delivery of cancer survivorship care, this study supports nurses to maximise their impact on cancer survivorship outcomes. To ensure ongoing relevance and effectiveness, the outlined capabilities should be regularly reviewed and updated to address evolving population needs and changes in cancer care. Additionally, further research is needed to evaluate the effectiveness of these capabilities and their impact on patient outcomes and healthcare resource utilisation and to adapt the framework to ensure equitable access to high quality, evidence-based cancer survivorship care across Australia.

## Supplementary information

Below is the link to the electronic supplementary material.

ESM 1 (DOCX 101 KB)

## Data Availability

The datasets generated and analysed during the current study are available from the corresponding author on reasonable request.
