# Supplementary material for: Quality cancer survivorship care: a modified Delphi study to define nurse capabilities
Source: J Cancer Surviv. 2025 May 3;20(Suppl 1):258–70. doi: 10.1007/s11764-025-01804-6 (PMC13676663; doi:10.1007/s11764-025-01804-6)
Supplement: Supplementary file 1 [file 11764_2025_1804_MOESM1_ESM.docx]

**Supplementary File 1: EdCaN Professional Development Model Definitions of nursing groups – All, Many, Some, Few**

Figure 1: EdCaN Professional Development Model for Nursing in Cancer Control Framework [1].

A description of the four broad groups of nurses in cancer control, including examples of scope of practice and associated competency standards are as follows:

**All nurses:** regardless of practice setting, are required to work collaboratively with the person affected by cancer to address their health needs. At all stages of life, from childhood to older age, and at several points across the cancer continuum, people affected by cancer will require services from nurses in generalist settings such as general practice, diagnostic services and general medical/surgical services. People affected by cancer may also have co-morbidities and may live with the consequences of cancer beyond an active diagnostic and treatment phase, in survivorship or at end of life. When in contact with people affected by cancer, all nurses need to be capable of applying generic nursing competencies to meet the health needs of these individuals. For example, some of the key cancer-care concepts identified as relevant for nurses entering practice include beginning-level skills in communication, psychological, social and emotional support and conceptualisation of the meaning of cancer [2,3].

**Many nurses**: will participate more frequently or for short intensive periods in the care of people affected by cancer due to their expertise in addressing specific health needs or because of their work context. Although not identified as cancer specialists, some of these nurses will be specialists in areas such as head and neck surgery, infection control, entero-stomal therapy, general paediatrics, or palliative care. Such nurses may also work in community health or rural and remote settings where they frequently come into contact with people affected by cancer. These nurses will demonstrate the application of core capabilities at a more advanced level in the particular cancer control contexts in which they practise. They will require access to further education in areas of specialist cancer control with a direct application to their role. Many of these nurses will also be able to demonstrate competence in one or more of the competencies outlined for the specialist cancer nurse, however the predominant focus of their practice is not within specialist cancer control.

**Some nurses:** will choose to become specialists in cancer control. Most specialist cancer nurses work in dedicated cancer services and may be primarily responsible for care of people at a specific phase of their journey (for example, radiotherapy), or across all phases of the cancer journey (for example, specialist breast nursing or paediatric oncology nursing). Others may work in a broader context but provide a specialist resource in cancer control to a range of generalist providers (for example, a cancer nurse coordinator). This Framework specifies a set of competency standards that reflect the specialised knowledge and skills required to provide safe and competent care to people affected by cancer. These standards have drawn on the competency standards for specialist breast nurses developed by the National Breast Cancer Centre[4], and are consistent with the generic competency standards for advanced nurses established by the Australian Nursing Federation [5]. The specialist cancer nurse competencies are a minimum standard. It is expected that as their practice advances, specialist cancer nurses demonstrate more effective integration of theory, practice and experience along with increasing degrees of autonomy in judgments and interventions for people affected by cancer [6]. There is currently no accepted national educational standard for specialist cancer nurses, but development of competency standards such as those specified in the EdCAN Framework would normally require further education in cancer control at the postgraduate level.

**Few nurses:** will become competent and authorised to practise in an advanced and/or extended role in cancer control, including those licensed to practise as a nurse practitioner. These nurses will build on the competencies of the specialist cancer nurse through additional experience and education at the master-degree level or equivalent. The practice of nurses in this group reflects a more advanced application of the competency standards for the specialist cancer nurse. For nurse practitioners in cancer control, using the ANMC’s competency standards as a framework, the application to cancer control settings can be used as a guide in determining scope of practice [7].

**References:**

1.	Aranda S, Yates P. A national professional development framework for cancer nursing. 2nd edn. Canberra: The National Cancer Nursing Education Project (EdCaN), Cancer Australia; 2009.

2.	Hicks C, Fide J. The educational needs of non-specialist breast care nurses. Nurse Educ Today. 2003;23(7):509-21.

3.	Cunningham SM, Copp G, Collins B, Bater M. Pre-registration nursing students' experience of caring for cancer patients. Eur J Oncol Nurs. 2006;10(1):59-67.

4.	National Breast Cancer Centre (NBCC). Specialist breast nurse competency standards and associated educational requirements. Camperdown, NSW: NBCC; 2005.

5.	Australian Nursing Federation (ANF). Competency standards for the advanced registered nurse. A Adrian (ed). Melbourne: ANF; 2006.

6.	Queensland Nursing Council (QNC). Scope of practice framework for nurses and midwives. Brisbane: QNC; 2005.

7.	Australian Nursing and Midwifery Council (ANMC). National competency standards for the nurse practitioner. Deakin, ACT: ANMC; 2006.
